# Prediction of methotrexate efficacy and adverse events in patients with juvenile idiopathic arthritis: a systematic literature review

**DOI:** 10.1186/1546-0096-12-51

**Published:** 2014-12-11

**Authors:** EH Pieter van Dijkhuizen, Nico M Wulffraat

**Affiliations:** Department of Paediatric Immunology, University Medical Centre Utrecht, Wilhelmina Children’s Hospital, Utrecht, The Netherlands; Pediatria II, Reumatologia, IRCCS G. Gaslini, Largo Gaslini, 5, 16147 Genova, Italy

**Keywords:** Juvenile idiopathic arthritis, Methotrexate, EFficacy, Efficaciousness, Adverse events, MTX intolerance, Prediction

## Abstract

**Background:**

Methotrexate (MTX) is the cornerstone disease-modifying anti-rheumatic drug in juvenile idiopathic arthritis (JIA). In JIA, it is important to start effective treatment early to avoid long-term sequelae, such as joint damage. To accomplish this goal, it is crucial to know beforehand who is going to respond well to MTX. In addition, MTX adverse effects such as MTX intolerance occur frequently, potentially hindering its efficacy. To avoid inefficacy of an otherwise effective drug, the physician should be timely aware of these adverse events. Consequently, to optimise treatment of JIA patients with MTX, predictors for efficacy and adverse events should be used in daily clinical practice. The aim of this study was to summarise the existing knowledge about such predictors.

**Methods:**

A systematic literature search was performed in PubMed, Embase and The Cochrane Library, and 1,331 articles were identified. These were selected based on their relevance to the topic and critically appraised according to pre-defined criteria. Predictors for MTX efficacy and adverse events were extracted from the literature and tabulated.

**Results:**

Twenty articles were selected. The overall quality of the studies was good. For MTX efficacy, candidate predictors were antinuclear antibody positivity, the childhood health assessment questionnaire score, the myeloid-related protein 8/14 level, long-chain MTX polyglutamates, bilateral wrist involvement and some single nucleotide polymorphisms (SNPs) in the adenosine triphosphate binding cassette and solute carrier transporter gene families. For MTX adverse events, potential predictors were alanine aminotransferase and thrombocyte level and two SNPs in the γ-glutamyl hydrolase and methylenetetrahydrofolate reductase genes. However, validation of most predictors in independent cohorts was still lacking.

**Conclusions:**

Interesting candidate predictors were found, especially for MTX efficacy. However, most of these were not validated. This should be the goal of future efforts. A clinically relevant way to validate the predictors is by means of creating a clinical prediction model.

**Electronic supplementary material:**

The online version of this article (doi:10.1186/1546-0096-12-51) contains supplementary material, which is available to authorized users.

## Background

Juvenile Idiopathic Arthritis (JIA) is the most common childhood rheumatologic disorder, with a prevalence of 16–150 per 100,000 children. It is characterised by chronic arthritis of unknown aetiology, lasting at least 6 weeks, with an onset before 16 years of age [1]. JIA is a heterogeneous group of disorders, whose manifestations range from relatively mild inflammation of a single joint, to severe involvement of multiple joints lasting into adulthood and leading to structural joint damage and incapacity. These long-term sequelae should be avoided and it is thought that early and effective therapy in the so-called window of opportunity is crucial in doing so [2–4].

The most widely used disease-modifying anti-rheumatic drug (DMARD) in the treatment of JIA is methotrexate (MTX), which has been used for more than 25 years. It is an inexpensive and safe drug and is beneficial in around 70% of JIA patients [5, 6]. Other treatment options include intra-articular joint injections or the more potent biologicals for MTX or corticosteroid resistant cases. It is still impossible to predict the individual prognosis and hence the treatment requirements at the onset of the disease [7], leading to the current step-up approach of starting MTX and adding a biological if the patient does not respond sufficiently well to MTX monotherapy. However, given the abovementioned goal to start effective treatment immediately in order to prevent joint damage and the fact that MTX monotherapy is completely ineffective in around 30% of patients, it is essential to know beforehand who is going to respond well to MTX and who is not. The latter group may then be prescribed a biological from the outset.

Next to drug effectiveness, its side effects should be taken into account. It has been shown previously that MTX despite being safe frequently causes transient elevation of liver enzymes and potentially also cytopenias, for which periodic evaluation of blood counts and liver function tests are advised [6, 8]. Perhaps more importantly, gastrointestinal side effects and MTX intolerance occur frequently [9–12]. MTX intolerance has been shown to influence the quality of life of patients negatively [13]. Furthermore, these adverse effects potentially cause non-compliance and hence ineffectiveness of an otherwise effective drug [9, 12, 14, 15], interfering with the goal to induce early disease remission. To avoid this problem, the risk of occurrence of these adverse effects should be known early, in order for the physician to intervene timely.

Therefore, to optimise treatment of JIA patients, it is necessary to predict the probability of response as well as the risk of developing adverse events. This systematic literature review aims to find and summarise studies, which assessed factors capable of doing so.

## Methods

**Figure 1:**
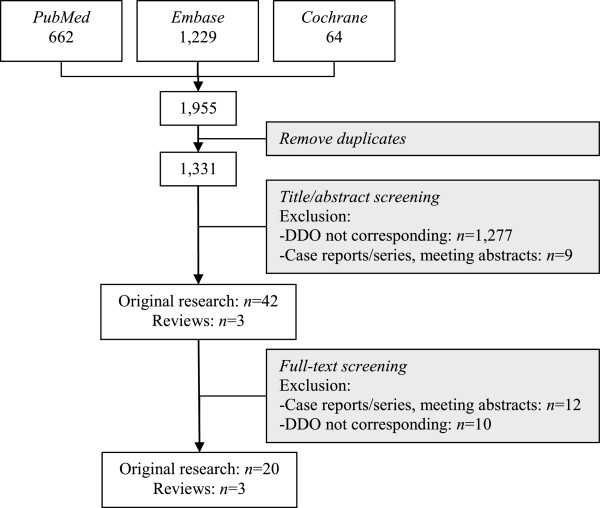
**Flow chart.** Flow chart of the article selection procedure. Abbreviations: DDO, domain, determinant and outcome.

On 20 April 2014, a systematic literature search was performed in PubMed, Embase and The Cochrane Library, without any publication date or language constraints. Using the algorithm in Table 1 to retrieve all papers regarding JIA and MTX, 1,331 articles were identified (Figure 1). These were then screened for applicability to the research subject, the identification of predictors of MTX efficacy and adverse effects (outcome) in JIA patients (domain). Based on the title and the abstract, 45 articles were selected for full-text screening (Figure 1). To ensure that all relevant articles had been found, references of selected articles were screened to identify any missed papers.

**Table 1:** **Search strategy** ^**a**^

	Search algorithm	PubMed ^b^	Embase ^b^	Cochrane ^c^
#1	“Juvenile idiopathic arthritis” OR “juvenile chronic arthritis” OR “juvenile rheumatoid arthritis” OR “juvenile rheumatic arthritis” OR “childhood arthritis” OR “juvenile arthritis” OR JIA OR JCA OR JRA	7,844	10,906	296
#2	Methotrexate OR MTX OR “disease-modifying antirheumatic drug” OR “disease-modifying antirheumatic drugs” OR “disease-modifying anti rheumatic drug” OR “disease-modifying anti rheumatic drugs” OR DMARD OR DMARDs	34,919	50,157	5,251
#3	#1 AND #2	662	1,229	64

^**a**^Search performed on 20 April 2014.

^**b**^In PubMed and Embase terms were searched in title and abstract only.

^**c**^In The Cochrane Library terms were searched in title, abstract and keywords only.

Selected articles were critically appraised, using predefined criteria (Table 2). Studies that were selected, aimed to find predictors for MTX efficacy or side effects within 6 months after the start of therapy in JIA patients, using standardised outcome criteria.

**Table 2:** **Critical appraisal**

Reference	Design	Relevance			Validity				
		Dom	Det	Out	Blind	Rec	SoC	Loss	Mis
*Outcome: MTX efficacy*									
[16]	Retrospective (validation prospective)	+	+	+/−	+	+	+	+	+
[17]	Mixed retrospective and prospective	+	+	+/−	+	+	+	+	+
[18]	Prospective	+	+	+/−	+	+	+	+	?
[19]	Prospective	+	+	+/−	+/−	+	?	?	+
[20]	Cross-sectional	+	+	+/−	+/−	+	?	?	+
[10]	Retrospective	+	+	+/−	?	+	?	+	+
[21]	Retrospective	+	+	+/−	?	+	?	?	+
[22]	Mixed retrospective and prospective	+	+/−	+/−	?	+	?	+	+
[23]	Prospective (validation unknown)	+	+/−	+/−	?	+	?	?	?
[24]	Prospective (validation unknown)	+	+/−	+/−	?	+	?	?	?
[25] ^**a**^	Retrospective	+/−	+/−	+	?	+	?	+/−	+
[26]	Prospective	+/−	+	+/−	?	+	?	+	+
[27]	Prospective	+/−	+	+/−	?	+	?	+	+/−
[28]	Prospective	+/−	+	+/−	?	+	?	+	?
[29]	Retrospective	+/−	+/−	+	?	+	?	+/−	+
[25] ^**b**^	Retrospective	+/−	+/−	+/−	?	+	?	+/−	+
[30]	**Retrospective**	**+**	**+**	**-**	**?**	**+**	**?**	**+**	**+**
[31]	**Cross-sectional**	**+**	**+**	**-**	**+/−**	**+**	**?**	**?**	**?**
[32]	**Retrospective**	**+**	**+/−**	**-**	**+/−**	**+**	**?**	**?**	**?**
[33]	**Retrospective**	**+/−**	**+**	**-**	**?**	**+**	**?**	**?**	**+**
*Outcome: MTX adverse effects*									
[34]	Prospective	+	+	+/−	+	+	+	+	+
[18]	Prospective	+	+	+/−	+	+	+	?	?
[10]	Retrospective	+	+	+/−	?	+	?	+	+
[21]	Retrospective	+	+	+/−	?	+	?	?	+
[20]	Cross-sectional	+	+	+/−	+/−	+	?	?	+
[31]	Cross-sectional	+	+	+/−	+/−	+	?	?	?
[32]	Retrospective	+	+/−	+/−	+/−	+	?	?	?
[35]	**Cross-sectional**	**-**	**+**	**+**	**?**	**-**	**?**	**+**	**+**

*Abbreviations: Blind* blinding, *Det* determinant, *Dom* domain, *Loss* loss to follow up, *Mis* missing predictors, *Ou*t outcome, *Rec* recruitment, *SoC* standardization of care.

Bold articles were excluded for analysis.

^**a**^Predictors after 6 months for outcome after 5 years; ^**b**^Predictors at baseline for outcome after 6 months.

**Criteria: Domain:** + Children with confirmed JIA, according to currently valid ILAR criteria, starting MTX +/− Children with JCA/JRA according to previously valid criteria, or children with JIA and additional criteria (e.g. hospitalized, specific categories only), starting MTX - Children without JIA/JCA/JRA, or no MTX; **Determinant:** + Prediction model or single predictors corrected for confounding in multivariable analysis +/− Single predictors in univariate analysis - No predictors; **Outcome:** + Efficacy: Any standardized outcome measurement, follow up >1 year. Adverse effects: Any outcome measurement, follow up >1 year +/− follow-up <1 year - Efficacy: No use of standardized outcome criteria; **Blinding:** + Both patient and physician blinded (or not applicable in case of objective measurements) +/− Patient or physician not blinded - Not blinded; **Recruitment:** + Predictors determined at time of start of MTX or <6 months (or time of determination does not matter as in genetic evaluations, gender, age at onset, etc.) +/− Predictors determined more than 6 months after start of MTX, but <1 year - Predictors determined after 1 year, or completely at random; **Standardization of care:** + All participants treated according to standards of care - No standardized care; **Loss to follow up (missing outcome):** + <20% and unselective loss to follow up; or >20%, unselective and solved with a statistically valid method (imputation) +/− >20% (not imputed) but unselective loss to follow up - Selective loss to follow up; **Missing predictors:** + <20% and unselective; or >20%, unselective and solved with a statistically valid method (imputation) +/− >20% (not imputed) but unselective - Selective missing predictors.

The assessed predictors in the selected studies were summarised in tabular form. Because of the high number of studies and predictors, it was decided to show the direction of the effect of each predictor only, instead of providing an odds ratio and 95% confidence interval. A cut point of P <0.05 was defined to denote significance. Even though some of the assessed studies aimed at constructing a prediction model, for which this cut point is not important, we report significant predictors (P <0.05) only.

## Results

After full-text screening, 20 original research papers and 3 reviews were selected (Figure 1), of which the former were critically appraised (Table 2). The overall relevance and validity of the selected papers was good, leading to only a few papers being excluded from the analysis. No study described whether all patients were eligible to receive the same treatment, but we knew this was the case in our own studies and assumed it was the case in all other studies. Hardly any article described if the physician and researchers were blinded for the outcome at the time of predictor determinations. Follow up was only short term (<1 year) in almost all studies.

### MTX efficacy

Fifteen studies assessed MTX efficacy, of which some used a derivation cohort and an independent replication cohort. Since these were independent cohorts, results obtained in these cohorts were reported as if obtained in separate studies. In most of the studies, MTX was started within a median time of 1.5 years after disease onset. The most often used outcome criteria were the American College of Rheumatology (ACR) response criteria. Follow up ranged from 6 months to 1 year, but was as much as 7.3 years in one study (Table 3).

**Table 3:** **Characteristics of included studies**

Reference	Design	Country of origin	***N***	Inclusion criteria	Outcome ^a^	Follow up
[18] ^**b**^	Prospective	The Netherlands	113	JIA, starting MTX	1k, 2a, 2d, 2f	1 y
[19]	Prospective	UK	87	JIA, starting MTX	1b, 1j	6 mo
[17] ^**c**^	Retrospective and prospective	The Netherlands	287	JIA, starting MTX	1c	1 y
[10]	Retrospective	Germany	411	JIA, starting MTX	1a, 1b, 1c, 2i	1 y
[16] (deriv)	Retrospective	The Netherlands	183	JIA, starting MTX	1e	1 y
[16] (rep)	Prospective	The Netherlands	104	JIA, starting MTX	1e	1 y
[23] (deriv)^**d**^	Prospective	UK	197	JIA, starting MTX	1d	6 mo
[23] (rep)^**d**^	Unknown	USA	210	JIA, starting MTX	1g	6 mo
[31]	Cross-sectional	Japan	92	JIA, at least 3 mo MTX	2e	Mean 58.2 mo^**e**^
[24] (deriv)^**d**^	Prospective	UK	197	JIA, starting MTX	1d	6 mo
[24] (rep)^**d**^	Unknown	USA	210	JIA, starting MTX	1g	6 mo
[20]	Cross-sectional	Czech Republic	69	JIA, at least 3 mo MTX	1i, 2d, 2f, 2g, 2h	Median 1.3-1.4 y^**e**^
[28] ^**f**^	Prospective	Multinational (PRINTO)	563	RF negative polyarticular course JIA, starting MTX	1a, 1c	6 mo
[26]	Prospective	Italy	60	JIA, ≥2 active joints in oligo persistent, ≥5 active joints in other categories	1b	1 y
[27] ^**f**^	Prospective	Multinational (PRINTO)	521	RF negative polyarticular course JIA, starting MTX	1l	6 mo
[25]	Retrospective	Italy	125	Polyarticular JIA, starting MTX	1f, 1i	6 mo, 5 y
[32]	Retrospective	Germany	58	JIA, at least 3 mo MTX	2d, 2i	Mean 48 months
[21]	Retrospective	Italy	80	JIA, at least 6 mo MTX	1a, 2c, 2g	Efficacy: 6 mo
						Toxicity: median 6–9 mo
[29]	Retrospective	USA	49	JRA, starting MTX	1h	Mean 2.6 y (range 1.0-7.3 y)
[34] ^**b**^	Prospective	The Netherlands	152	JIA, starting MTX	2b	1 y
[22]	Retrospective and prospective	Czech Republic, UK, The Netherlands	694	JIA, starting MTX	1f	6 mo

*Abbreviations: ACR30/50/70* American College of Rheumatology pediatric 30, 50 or 70 response criteria, respectively, *AE* adverse event, *ALT* alanine aminotransferase, *AST* aspartate aminotransferase, *CHQ* child health questionnaire, *deriv* derivation cohort, *GI* gastrointestinal, *HRQOL* health-related quality of life, *JADAS* juvenile arthritis disease activity score, *JIA* juvenile idiopathic arthritis, *min* minutes, *MISS* methotrexate intolerance severity score, *mo* months, *MTX* methotrexate, *NR* non-response, *PhS* physical component summary score, *PsS* psychosocial component summary score, *RA* rheumatoid arthritis, *rep* replication cohort, *RF* rheumatoid factor, *ULN* upper limit of normal, *y* years.

^**a**^ **1a:** Achievement of ACR30; **1b:** Achievement of ACR50; **1c:** Achievement of ACR70; **1d:** Achievement of ACR70 vs. non-achievement of ACR30; **1e:** Achievement of ACR70 in 2/3 visits; **1f:** NR vs. ACR30 vs. ACR50 vs. ACR70; **1g:** >70% improvement in joint count vs. <30%; **1h:** Adapted ACR criteria for RA: morning stiffness <15 min, no fatigue, no joint swelling, no joint pain for 2 consecutive months; **1i:** Clinical inactive disease on MTX monotherapy according to Wallace criteria; **1j:** JADAS-10; **1k:** JADAS-27; **1l:** HRQOL: CHQ PhS ≥30 and PsS ≥30; **2a:** MISS: intolerant (score >6); **2b:** MISS: intolerant (score >6) after 6 and/or 12 months; **2c:** ALT/AST > ULN; **2d:** ALT/AST >2 ULN; **2e:** ALT >5 ULN; **2f:** Bone marrow suppression (any cytopenia); **2g:** GI toxicity; **2h:** Other (alopecia, headaches, behavioural changes, nodulosis); **2i:** Any AE; ^**b**^This is the same cohort as the replication cohort of [16], but different outcome and/or predictors; ^**c**^This cohort is the derivation and replication cohort of [16] together, but uses a slightly different outcome and different predictors; ^**d**^These are the same cohorts, but they use different predictors; ^**e**^Time after start of MTX; ^**f**^These are the same cohorts, but they use different outcome measurements.

The results of these studies are shown in Additional file 1: Table S1. Demographics, as well as JIA categories, were analysed extensively and were not predictive in almost all studies. Disease activity parameters showed inconsistent results in general, but the childhood health assessment questionnaire (CHAQ) score was a potential predictor. The same held true for the physician’s global assessment (PGA), although less convincingly so. The involvement of individual joints was assessed in too few studies to be conclusive, but bilateral wrist involvement was a potential predictor. Among laboratory data, positive antinuclear antibody (ANA) was a predictor of better response in three studies. Other interesting predictors could be long-chain MTX polyglutamates (PGs), the myeloid-related protein (MRP) 8/14 (also known as S100A8/A9), the pro-inflammatory molecule osteopontin, or even the haemoglobin level, although these were assessed in only one study each (Additional file 1: Table S1).

Next to these predictors, many single-nucleotide polymorphisms (SNPs) were analysed. These were SNPs in genes involved in the MTX metabolic pathway and in genes with altered post-treatment gene expression. Moreover, recently a genome-wide analysis study (GWAS) was published [22]. Of the latter study, only gene regions showing association with MTX response could be reported in this review.

Overall, no unequivocal predictive SNP has been found yet, because many were assessed in only one study, or were predictive in one study and showed no effect in others. However, some SNPs (rs1045642, rs35592 and rs4793665) in the B1, C1 and C3 members of the adenosine triphosphate binding cassette (*ABC*) transporter family, and others (rs3763980 and rs1051266) in the 16A7 and 19A1 members of the solute carrier (*SLC*) transporter family were interesting. Furthermore, the gene regions associated in the GWAS study were promising predictors (Additional file 1: Table S1).

In all, many potential predictors for MTX efficacy were assessed, yielding some interesting candidates, which, however, were analysed in too few studies to draw a firm conclusion yet.

### MTX adverse events

Seven of the selected studies assessed MTX adverse events (Table 3). The assessed outcome varied from overall adverse events to single adverse events such as liver toxicity, gastrointestinal complaints or MTX intolerance measured with the Methotrexate Intolerance Severity Score (MISS) [9]. None of the articles focused on (serious) infections. Since the outcome MTX adverse events is a composite of all these outcomes, all studies were included. Follow up ranged from 6 months to a mean of 58.2 months.

Many predictors were evaluated in only one or two studies, making the results inconclusive. However, interesting predictors were the alanine aminotransferase (ALT) and thrombocyte level, as well as a SNP (rs1800909) in the γ-glutamyl hydrolase (*GGH*) gene, involved in the breakdown of MTX PGs, and another SNP (rs1801133) in the methylenetetrahydrofolate reductase (*MTHFR*) gene, involved in the folate metabolism. Finally, the polyarticular categories could potentially pose a risk to develop MTX side effects (Additional file 2: Table S2).

## Discussion

This systematic literature review aimed to find predictors for MTX efficacy and adverse events in JIA patients. For MTX efficacy, many candidate predictors were investigated, and some interesting results were found, such as ANA positivity, the CHAQ score, the MRP 8/14 (S100A8/A9) level, long-chain MTX-PGs, bilateral wrist involvement, osteopontin level, haemoglobin, some SNPs in the *ABC* and *SLC* transporter gene families and several gene regions elucidated in the recently published GWAS. Most of these variables have not yet been validated in independent cohorts. Therefore, future efforts should be directed at validating these candidate predictors. A clinically relevant way to do so consists in combining these predictors into a prediction model and assessing the prognostic accuracy of the model. Thus far, only one prediction model for MTX efficacy in JIA patients has been developed, containing the erythrocyte sedimentation rate and four SNPs in genes involved in the MTX metabolic pathway [16]. This model could be improved using the abovementioned candidate predictors. The advantage of this method is twofold: statistically, the selection of predictors for the model in the independent cohort will be literature-driven, instead of data-driven, leading to a reduction in the so-called optimism [35]. Clinically, physicians will have an easy-to-use tool at hand to determine the probability of MTX efficacy in individual patients, allowing them to start MTX in patients with a high probability of responding and to initiate biologicals in those with a low probability of responding. The clinical benefit of this approach should ideally be estimated in a randomized clinical trial.

Regarding MTX adverse events, the results were less clear. Although some interesting candidate predictors were found, such as ALT and thrombocyte level and two SNPs in the *GGH* and *MTHFR* genes, here too, validation of these was lacking. Furthermore, it seemed questionable if these predictors would be sufficient to predict the individual patients’ risk of developing MTX adverse effects. Consequently, future efforts should be directed both at validating existing candidate predictors and at finding new predictors. These too should be combined in a clinical prediction model (an example has been submitted: Van Dijkhuizen EHP, Bulatovic Calasan M, Pluijm SMF, De Rotte MCFJ, Vastert SJ, Kamphuis S, De Jonge R, Wulffraat NM: Prediction of Methotrexate Intolerance in Juvenile Idiopathic Arthritis, submitted). Such a tool could be used to monitor high-risk patients more closely, intervening as soon as adverse events occur, for example by lowering the dose, stopping MTX temporarily or prescribing other drugs. On the other hand, low-risk patients could be saved the burden of frequent checks.

Recently, a review was published about genetic predictors of MTX efficacy and toxicity in rheumatoid arthritis [36]. SNPs investigated in five or more independent studies were considered (n = 4). Only *ATIC* rs2372536 showed a potential association of the minor allele with toxicity. This SNP did not show an association with adverse events in our review. Conversely, whereas we found an association of *SLC19A1* rs1051266 with efficacy (though in a single study), results about this SNP were inconsistent in the adult review. Thus, the results of the adult and paediatric review were quite different. This might be due to incomparability of children and adults in this respect, maybe because of a different metabolization of MTX [37]. On the other hand, it shows there is still much to do in the field of MTX prediction.

Most SNPs investigated in this review were located in genes involved in the MTX metabolic pathway. In short, MTX enters the cell via the members of the SLC protein family. It becomes polyglutamated by FPGS, causing its cellular retention. Depolyglutamation is brought about by GGH. MTX is pumped out of the cell by members of the ABC transporter family. Intracellular MTX-PGs exert a range of actions. First, they inhibit DHFR and influence MTHFR, enzymes in the folate pathway involved in polyamine synthesis. Secondly, they inhibit TYMS, an enzyme in the pyrimidine synthesis pathway. Finally, by blocking ATIC, MTX-PGs stimulate the production of adenosine, an anti-inflammatory agent. Other important enzymes in this pathway are ITPA and AMPD1, which themselves are not influenced by MTX [17, 36]. It can be hypothesized that SNPs in any of these genes cause increased or decreased sensibility to the actions of MTX-PGs and hence lead to altered MTX efficacy.

Other potential predictors included the MRP8/14 (S100A8/A9) level, a danger signal and activator of toll-like receptor 4, which in turn plays a role in the innate immune response in inflammatory conditions. MRP8/14 was earlier shown to predict disease flare or continuation of remission after withdrawal of MTX in children who were in remission [5]. Another candidate predictor, osteopontin, is expressed by natural killer cells and activated T cells, and plays a role in the production of pro-inflammatory cytokines. It is overexpressed in synovial T cells in patients with rheumatoid arthritis, demonstrating its role in inflammatory arthritis [26]. The theoretical background of these candidate predictors may increase the likelihood that they really affect MTX efficacy, however, it should be kept in mind that the found associations do not prove a causal effect.

The most frequently used outcome criteria with respect to MTX efficacy were the American College of Rheumatology (ACR) response criteria [38]. These criteria, though validated, have their limitations in practical use. For example, a patient with 69% improvement in all core set criteria will not be an ACR70 responder, whereas someone with 70% improvement in three core set criteria and worsening up to 29% in the remainder will be an ACR70 responder, despite the fact that the former obviously responds better than the latter. Hence, if one takes the achievement of ACR70 response status as an outcome measurement, considerable misclassification may occur. Alternatively, the percentage of change of the juvenile arthritis disease activity score (JADAS) could be used as an outcome measurement to assess MTX efficacy [39, 40]. Because this is a continuous and composite outcome, the risk of misclassification may be reduced. Recently, the ACR criteria and the JADAS were compared, showing excellent ability of the JADAS to classify the ACR response categories [41]. To answer the question which outcome measurement should be preferred, both measurements should be compared to a reference standard, such as a panel of experts.

The studies that were analysed in this review were generally of good quality. They recruited patients at the start of MTX and followed them prospectively. However, many articles did not describe whether the studies were blinded, an absence of which could lead to biases. On a review level, due to our extensive search strategy, it is likely that we found all pertinent papers. However, some negative results might not have been published, leading to reporting and publication bias. The results found in different studies were often quite variable. This may be due to heterogeneity of patient groups, differences in sample size and the absence or presence of linkage disequilibrium between the tested SNP and the actual polymorphism which causes the altered MTX response [36]. Furthermore, other patient factors may confound the observed relationship, reason why it is important to perform a multivariate analysis. This was not done in all studies. Finally, the authors of this review were co-authors of some of the included papers, causing them to know more about the study design and potentially biasing them to be more lenient towards their own studies.

## Conclusion

In conclusion, this systematic review of the literature with respect to predictors for MTX efficacy and adverse events in JIA patients shows that a number of interesting candidates were found. However, validation of these potential predictors is still lacking in many cases. Therefore, future efforts should be directed at validating these candidate predictors, potentially by means of a clinical prediction model. For the outcome adverse events, next to validating existing candidates, more candidate predictors should be investigated.

## Electronic supplementary material

Additional file 1: Table S1: Results for outcome MTX efficacy^a^. (DOCX 436 KB)

Additional file 2: Table S2: Results for outcome absence of MTX adverse events^a^. (DOCX 116 KB)
