# Supplementary material for: Prediction of methotrexate efficacy and adverse events in patients with juvenile idiopathic arthritis: a systematic literature review
Source: Pediatr Rheumatol Online J. 2014 Dec 11;12:51. doi: 10.1186/1546-0096-12-51 (PMC4269851; doi:10.1186/1546-0096-12-51)
Supplement: Supplementary file 1 — Additional file 1: Table S1: Results for outcome MTX efficacya. (DOCX 436 KB) [file 12969_2014_2173_MOESM1_ESM.docx]

| **Additional file 1: Table S1.** Results for outcome MTX efficacy**^a^** | | | | | | | |
| --- | --- | --- | --- | --- | --- | --- | --- |
| Baseline predictors (within 6 months after MTX start) | Number of studies | | | | | | References |
|  | -- | <**^b^** | NS | S**^b^** | >**^b^** | + |  |
| *Demographics* |  |  |  |  |  |  |  |
| Gender: female |  |  | 8 |  |  |  | [1-7] |
| Higher age at onset |  |  | 7 |  |  |  | [1,2,4-7] |
| Higher age at MTX start |  |  | 8 |  |  |  | [1-4,6-8] |
| Longer disease duration before MTX start | 1 |  | 7 |  |  |  | [1,2,4-8] |
| Higher body surface area |  |  | 1 |  |  |  | [3] |
|  |  |  |  |  |  |  |  |
| *JIA category****^c^*** |  |  |  |  |  |  |  |
| Oligoarticular persistent |  |  | 1 |  |  |  | [8] |
| Oligoarticular extended |  |  | 3 |  |  | 1 | [4-6,8] |
| Systemic |  |  | 7 |  |  |  | [1,4-8] |
| Polyarticular, rheumatoid factor negative |  | 0/1 | 6 |  |  |  | [1,4-8] |
| Polyarticular, rheumatoid factor positive |  | 0/1 | 6 |  |  |  | [1,4-8] |
| Psoriatic |  |  | 2 |  |  |  | [1] |
| Enthesitis-related arthritis |  |  | 2 |  |  |  | [1] |
| Undifferentiated |  |  | 2 |  |  |  | [1] |
|  |  |  |  |  |  |  |  |
| *Disease activity* |  |  |  |  |  |  |  |
| Higher active joint count |  | 1/0 | 5 | 1/0 | 1/0 |  | [1-7] |
| Higher swollen joint count |  | 1/0 |  | 1/0 |  |  | [6,7] |
| Higher limited joint count |  |  | 6 |  |  | 1 | [1-3,5-7] |
| Higher painful joint count |  |  | 1 | 1/0 |  |  | [6,7] |
| Higher PGA |  |  | 4 |  | 1/0 | 1 | [1,2,5-7] |
| Higher parent/patient GA | 1 |  | 2 |  |  | 1 | [2,5-7] |
| Higher pain VAS | 1 |  | 1 |  |  |  | [6,7] |
| Higher CHAQ score | 2 | 1/0 | 2 |  |  |  | [2,4-7] |
| Functional status |  |  | 1 |  |  |  | [8] |
| Higher CHQ PhS score |  |  | 1 |  |  |  | [7] |
| Higher CHQ PsS score |  |  | 1 |  |  |  | [7] |
| Achievement ACR70 at 6 months |  |  |  |  | 0/1 |  | [5] |
| Hand or wrist involvement |  | 1/0 |  |  |  |  | [4] |
| Wrist: right and left activity | 1 |  |  |  |  |  | [7] |
| Wrist: ≥1 active |  | 1/0 |  |  |  |  | [7] |
| MCP 1: right and left activity |  | 1/0 |  |  |  |  | [7] |
| MCP 1: ≥1 active |  | 1/0 |  |  |  |  | [7] |
| MCP 2: ≥1 active |  | 1/0 |  |  |  |  | [7] |
| MCP 3: ≥1 active |  | 1/0 |  |  |  |  | [7] |
| PIP 1: right and left activity |  | 1/0 |  |  |  |  | [7] |
| PIP 2: right and left activity |  | 1/0 |  |  |  |  | [7] |
| Knee: ≥1 active |  |  |  |  | 1/0 |  | [7] |
|  |  |  |  |  |  |  |  |
| *Laboratory* |  |  |  |  |  |  |  |
| ANA positive |  |  | 2 |  | 0/1 | 2 | [1,6-8] |
| Rheumatoid factor positive |  |  | 1 |  |  |  | [8] |
| Higher ESR | 1 |  | 5 |  | 2/1 |  | [1-7,9] |
| Higher CRP |  |  | 1 |  |  |  | [2] |
| Higher haemoglobin |  |  |  |  |  | 1 | [9] |
| Higher MRP8/14 |  |  |  |  |  | 1 | [2] |
| Higher IL-1β |  |  | 1 |  |  |  | [2] |
| Higher IL-2 |  |  |  |  | 1/0 |  | [2] |
| Higher IL-6 |  |  | 1 |  |  |  | [2] |
| Higher IL-10 |  |  | 1 |  |  |  | [2] |
| Higher IL-12 |  |  | 1 |  |  |  | [2] |
| Higher IL-18 |  |  | 1 |  |  |  | [2] |
| Higher IL-22 |  |  | 1 |  |  |  | [2] |
| Higher TNF-α |  |  | 1 |  |  |  | [2] |
| Higher IFN-γ |  |  | 1 |  |  |  | [2] |
| Higher osteopontin level | 1 |  |  |  |  |  | [9] |
|  |  |  |  |  |  |  |  |
| *Medication* |  |  |  |  |  |  |  |
| Higher MTX start dose |  |  | 6 |  |  |  | [1,2,5,7,8] |
| Subcutaneous route of administration |  |  | 3 |  |  |  | [2,7,10] |
| Start with folic acid |  |  | 2 |  |  |  | [1] |
| Taking corticosteroids |  |  | 2 |  |  |  | [4,7] |
| Taking NSAIDs |  |  | 1 |  |  |  | [7] |
|  |  |  |  |  |  |  |  |
| *MTX-polyglutamates* |  |  |  |  |  |  |  |
| Higher MTX-PG1 |  |  | 1 |  |  |  | [11] |
| Higher MTX-PG2 |  |  | 1 |  |  |  | [11] |
| Higher MTX-PG3 |  |  |  |  |  | 1 | [11] |
| Higher MTX-PG4 |  |  |  |  |  | 1 | [11] |
| Higher MTX-PG5 |  |  |  |  |  | 1 | [11] |
| Higher MTX-PG3-5 |  |  |  |  |  | 1 | [11] |
| Higher total MTX-PG |  |  | 1 |  |  |  | [11] |
|  |  |  |  |  |  |  |  |
| *Imaging* |  |  |  |  |  |  |  |
| Presence of radiologic lesions |  |  | 2 |  |  |  | [4,8] |
| Higher Poznanski score |  |  | 1 |  |  |  | [5] |
|  |  |  |  |  |  |  |  |
| *Genetics****^d^*** |  |  |  |  |  |  |  |
| *MTHFR* rs7538516, minor allele |  |  | 1 |  |  |  | [12] |
| *MTHFR* rs4846047, minor allele |  |  | 1 |  |  |  | [12] |
| *MTHFR* rs4846049, minor allele |  |  | 1 |  |  |  | [12] |
| *MTHFR* rs2274976, minor allele |  |  | 1 |  |  |  | [12] |
| *MTHFR* rs3818762, minor allele |  |  | 1 |  |  |  | [12] |
| *MTHFR* rs12121543, minor allele |  |  | 1 |  |  |  | [12] |
| *MTHFR* rs1801133, dominant model |  |  | 3 |  |  |  | [1,3] |
| *MTHFR* rs1801133, homozygous variant vs. wild type |  |  | 1 |  |  |  | [3] |
| *MTHFR* rs1801133, heterozygous vs. wild type |  |  | 1 |  |  |  | [3] |
| *MTHFR* rs1801133, minor allele |  |  | 2 |  |  |  | [3,12] |
| *MTHFR* rs1801131, dominant model |  |  | 2 |  | 0/1 |  | [1,3] |
| *MTHFR* rs1801131, homozygous variant vs. wild type |  |  | 1 |  |  |  | [3] |
| *MTHFR* rs1801131, heterozygous vs. wild type |  |  | 1 |  |  |  | [3] |
| *MTHFR* rs1801131, minor allele |  |  | 1 |  |  |  | [3] |
| *MTHFR* rs17367504, minor allele |  |  | 1 |  |  |  | [12] |
| *MTHFR* rs7553194, minor allele |  |  | 1 |  |  |  | [12] |
| *MTRR* rs1801394, dominant model |  |  | 1 |  | 1/0 |  | [1] |
| *AMPD1* rs6658815, minor allele |  |  | 1 |  |  |  | [12] |
| *AMPD1* rs6679869, minor allele |  |  | 1 |  |  |  | [12] |
| *AMPD1* rs2268699, minor allele |  |  | 1 |  |  |  | [12] |
| *AMPD1* rs2336363, minor allele |  |  | 1 |  |  |  | [12] |
| *AMPD1* rs2268701, minor allele |  |  | 1 |  |  |  | [12] |
| *AMPD1* rs17602729, dominant model |  |  | 2 |  |  |  | [1] |
| *AMPD1* rs17602729, minor allele |  |  | 1 |  |  |  | [12] |
| *AMPD1* rs11587596, minor allele |  |  | 1 |  |  |  | [12] |
| *ATIC* rs2372536, dominant model |  |  | 2 |  |  |  | [1] |
| *ATIC* rs12995526, minor allele |  | 0/1 | 1 |  |  |  | [12] |
| *ATIC* rs12477799, minor allele |  |  | 1 |  |  |  | [12] |
| *ATIC* rs16853826, minor allele |  |  | 1 |  |  |  | [12] |
| *ATIC* rs4673990, minor allele |  | 0/1 | 1 |  |  |  | [12] |
| *ATIC* rs4672768, minor allele |  |  | 1 |  |  |  | [12] |
| *ATIC* rs10498036, minor allele |  |  | 1 |  |  |  | [12] |
| *ABCB1* rs1128503, recessive model |  |  | 3 |  |  |  | [1,13] |
| *ABCB1* rs1045642, recessive model |  |  | 1 |  | 1/0 | 1 | [1,13] |
| *ABCB1* rs2032582, recessive model |  |  | 1 |  |  |  | [13] |
| *ABCB1* rs1128503/rs32032582/rs1045642 haplotype AAA, recessive model |  |  | 1 |  |  |  | [13] |
| *ABCB1* rs1128503/rs32032582/rs1045642 haplotype GCG, recessive model |  | 1/0 |  |  |  |  | [13] |
| *ABCC1* rs35592, dominant model |  |  | 1 |  |  | 1 | [1] |
| *ABCC1* rs35592, recessive model |  |  |  |  | 1/0 |  | [13] |
| *ABCC1* rs3784862, dominant model |  |  | 2 |  |  |  | [1] |
| *ABCC1* rs3784862, recessive model |  |  | 1 |  |  |  | [13] |
| *ABCC2* rs4148396, recessive model |  |  | 1 |  |  |  | [13] |
| *ABCC2* rs717620, dominant model |  |  | 2 |  |  |  | [1] |
| *ABCC2* rs717620, recessive model |  |  | 1 |  |  |  | [13] |
| *ABCC2* rs4148396/rs717620 haplotype TC, recessive model |  |  | 1 |  |  |  | [13] |
| *ABCC2* rs4148396/rs717620 haplotype TT, recessive model |  |  | 1 |  |  |  | [13] |
| *ABCC3* rs4793665, dominant model |  |  | 2 |  |  |  | [1] |
| *ABCC3* rs4793665, recessive model |  |  |  |  |  | 1 | [13] |
| *ABCC3* rs3785911, dominant model |  |  | 2 |  |  |  | [1] |
| *ABCC3* rs3785911, recessive model |  |  | 1 |  |  |  | [13] |
| *ABCC5* rs2139560, dominant model |  |  | 2 |  |  |  | [1] |
| *ABCC5* rs2139560, recessive model |  |  | 1 |  |  |  | [13] |
| *ABCG2* rs13120400, dominant model |  |  | 2 |  |  |  | [1] |
| *ABCG2* rs13120400, recessive model |  | 1/0 |  |  |  |  | [13] |
| *ABCG2* rs13120400/rs2231142 haplotype CC, recessive model |  |  | 1 |  |  |  | [13] |
| *ABCG2* rs13120400/rs2231142 haplotype CG, recessive model |  | 1/0 |  |  |  |  | [13] |
| *ABCG2* rs13120400/rs2231142 haplotype TG, recessive model |  |  |  |  | 1/0 |  | [13] |
| *ABCG2* rs2728124, minor allele |  |  | 1 |  |  |  | [12] |
| *ABCG2* rs2231164, minor allele |  |  | 1 |  |  |  | [12] |
| *ABCG2* rs12505410, minor allele |  |  | 1 |  |  |  | [12] |
| *ABCG2* rs2622621, minor allele |  |  | 1 |  |  |  | [12] |
| *ABCG2* rs2199936, minor allele |  |  | 1 |  |  |  | [12] |
| *ABCG2* rs1564481, minor allele |  |  | 1 |  |  |  | [12] |
| *ABCG2* rs3114018, minor allele |  |  | 1 |  |  |  | [12] |
| *ABCG2* rs3109823, minor allele |  |  | 1 |  |  |  | [12] |
| *ABCG2* rs6857600, minor allele |  |  | 1 |  |  |  | [12] |
| *ABCG2* rs2622626, minor allele |  |  | 1 |  |  |  | [12] |
| *ABCG2* rs17731799, minor allele |  |  | 1 |  |  |  | [12] |
| *DHFR* rs1222809, minor allele |  |  | 1 |  |  |  | [12] |
| *DHFR* rs12517451, minor allele |  |  | 1 |  |  |  | [12] |
| *DHFR* rs1650723, minor allele |  |  | 1 |  |  |  | [12] |
| *DHFR* rs1643657, minor allele |  |  | 1 |  |  |  | [12] |
| *DHFR* rs11951910, minor allele |  |  | 1 |  |  |  | [12] |
| *DHFR* rs10072026, minor allele |  |  | 1 |  |  |  | [12] |
| *DHFR* rs380691, minor allele |  |  | 1 |  |  |  | [12] |
| *GGH* rs11545078, minor allele |  |  | 1 |  |  |  | [12] |
| *GGH* rs12335094, minor allele |  |  | 1 |  |  |  | [12] |
| *GGH* rs16930092, minor allele |  |  | 1 |  |  |  | [12] |
| *GGH* rs3780130, minor allele |  |  | 1 |  |  |  | [12] |
| *GGH* rs10957267, minor allele |  |  | 1 |  |  |  | [12] |
| *GGH* rs17194931, minor allele |  |  | 1 |  |  |  | [12] |
| *GGH* rs719235, minor allele |  |  | 1 |  |  |  | [12] |
| *GGH* rs10106587, dominant model |  |  | 2 |  |  |  | [1] |
| *GGH* rs10106587, recessive model |  |  | 1 |  |  |  | [13] |
| *GGH* rs3758149, dominant model |  |  | 2 |  |  |  | [1] |
| *GGH* rs3758149, recessive model |  |  | 1 |  |  |  | [13] |
| *GGH* rs3758149, minor allele |  |  | 1 |  |  |  | [12] |
| *GGH* rs10106587/rs3758149 haplotype AA, recessive model |  |  | 1 |  |  |  | [13] |
| *GGH* rs10106587/rs3758149 haplotype CG, recessive model |  |  | 1 |  |  |  | [13] |
| *GGH* rs10106587/rs3758149 haplotype AG, recessive model |  |  | 1 |  |  |  | [13] |
| *GGH* rs6998134, minor allele |  |  | 1 |  |  |  | [12] |
| *FPGS* rs4451422, recessive model |  |  | 1 |  |  |  | [13] |
| *FPGS* rs4451422, minor allele |  |  | 1 |  |  |  | [12] |
| *FPGS* rs10819309, minor allele |  |  | 1 |  |  |  | [12] |
| *MTHFD1* rs2983733, minor allele |  |  | 1 |  |  |  | [12] |
| *MTHFD1* rs1956545, minor allele |  |  | 1 |  |  |  | [12] |
| *MTHFD1* rs8011839, minor allele |  |  | 1 |  |  |  | [12] |
| *MTHFD1* rs8003379, minor allele |  |  | 1 |  |  |  | [12] |
| *MTHFD1* rs1950902, minor allele |  |  | 1 |  |  |  | [12] |
| *MTHFD1* rs17101851, minor allele |  |  | 1 |  |  |  | [12] |
| *MTHFD1* rs3783726, minor allele |  |  | 1 |  |  |  | [12] |
| *MTHFD1* rs2236224, minor allele |  |  | 1 |  |  |  | [12] |
| *MTHFD1* rs11629135, minor allele |  |  | 1 |  |  |  | [12] |
| *MTHFD1* rs3818239, minor allele |  |  | 1 |  |  |  | [12] |
| *MTHFD1* rs1256146, minor allele |  |  | 1 |  |  |  | [12] |
| *MTHFD1* rs11627387, minor allele |  |  | 1 |  |  |  | [12] |
| *MTHFD1* rs745686, minor allele |  |  | 1 |  |  |  | [12] |
| *MTHFD1* rs3742609, minor allele |  |  | 1 |  |  |  | [12] |
| *SHMT1* rs1979276, minor allele |  |  | 1 |  |  |  | [12] |
| *SHMT1* rs2168781, minor allele |  |  | 1 |  |  |  | [12] |
| *SHMT1* rs11868708, minor allele |  |  | 1 |  |  |  | [12] |
| *SHMT1* rs2273027, minor allele |  |  | 1 |  |  |  | [12] |
| *SHMT1* rs2273026, minor allele |  |  | 1 |  |  |  | [12] |
| *SHMT1* rs9901160, minor allele |  |  | 1 |  |  |  | [12] |
| *SHMT1* rs8065874, minor allele |  |  | 1 |  |  |  | [12] |
| *TYMS* rs9966612, minor allele |  |  | 1 |  |  |  | [12] |
| *TYMS* rs11664283, minor allele |  |  | 1 |  |  |  | [12] |
| *TYMS* rs2853741, minor allele |  |  | 1 |  |  |  | [12] |
| *TYMS* rs2853533, minor allele |  |  | 1 |  |  |  | [12] |
| *TYMS* rs2612095, minor allele |  |  | 1 |  |  |  | [12] |
| *TYMS* rs2847150, minor allele |  |  | 1 |  |  |  | [12] |
| *TYMS* rs9948583, minor allele |  |  | 1 |  |  |  | [12] |
| *TYMS* rs2298582, minor allele |  |  | 1 |  |  |  | [12] |
| *TYMS* rs2741186, minor allele |  |  | 1 |  |  |  | [12] |
| *TYMS* rs7239738, minor allele |  |  | 1 |  |  |  | [12] |
| *ITPA* rs6051639, minor allele |  |  | 1 |  |  |  | [12] |
| *ITPA* rs2295553, minor allele |  | 0/1 |  |  |  |  | [12] |
| *ITPA* rs6051644, minor allele |  |  | 1 |  |  |  | [12] |
| *ITPA* rs6084305, minor allele |  |  | 1 |  |  |  | [12] |
| *ITPA* rs1127354, minor allele |  |  | 1 |  |  |  | [12] |
| *ITPA* rs4815576, minor allele |  |  | 1 |  |  |  | [12] |
| *ITPA* rs6037506, minor allele |  |  | 1 |  |  |  | [12] |
| *ITPA* rs6139035, minor allele |  |  | 1 |  |  |  | [12] |
| *ITPA* rs13830, minor allele |  |  | 1 |  |  |  | [12] |
| *ITPA* rs6051655, minor allele |  |  | 1 |  |  |  | [12] |
| *ITPA* rs3810560, minor allele |  |  | 1 |  |  |  | [12] |
| *SLC16A7* rs17122830, minor allele |  |  | 2 |  |  |  | [14] |
| *SLC16A7* rs1497474, minor allele |  |  | 2 |  |  |  | [14] |
| *SLC16A7* rs10877327, minor allele |  |  | 1 |  |  |  | [14] |
| *SLC16A7* rs7976956, minor allele |  |  | 1 |  |  |  | [14] |
| *SLC16A7* rs1000708, minor allele |  |  | 1 |  |  |  | [14] |
| *SLC16A7* rs7971953, minor allele |  |  | 2 |  |  |  | [14] |
| *SLC16A7* rs12231740, minor allele |  |  | 2 |  |  |  | [14] |
| *SLC16A7* rs2711669, minor allele |  |  | 2 |  |  |  | [14] |
| *SLC16A7* rs2706301, minor allele |  |  | 1 |  |  |  | [14] |
| *SLC16A7* rs10877333, minor allele |  |  | 1 |  | 0/1 |  | [14] |
| *SLC16A7* rs2711655, minor allele |  | 0/1 |  |  |  |  | [14] |
| *SLC16A7* rs12718000, minor allele |  |  | 1 |  |  |  | [14] |
| *SLC16A7* rs3763980, minor allele |  | 0/2 |  |  |  |  | [14] |
| *SLC16A7* rs10784000, minor allele |  |  | 1 |  |  |  | [14] |
| *SLC16A7* rs2706301/ rs10877333/ rs2711655/ rs12718000/ rs3763980/ rs10784000 haplotype GTGGAA |  | 0/1 |  |  |  |  | [14] |
| *SLC16A7* rs2706301/ rs10877333/ rs2711655/ rs12718000/ rs3763980/ rs10784000 haplotype CTAGTG |  |  | 1 |  |  |  | [14] |
| *SLC16A7* rs2706301/ rs10877333/ rs2711655/ rs12718000/ rs3763980/ rs10784000 haplotype GGAGTA |  |  |  |  | 0/1 |  | [14] |
| *SLC16A7* rs2706301/ rs10877333/ rs2711655/ rs12718000/ rs3763980/ rs10784000 haplotype CTAGTA |  |  | 1 |  |  |  | [14] |
| *SLC16A7* rs2706301/ rs10877333/ rs2711655/ rs12718000/ rs3763980/ rs10784000 haplotype GTGATA |  |  | 1 |  |  |  | [14] |
| *SLC16A7* rs2706301/ rs10877333/ rs2711655/ rs12718000/ rs3763980/ rs10784000 haplotype GTGGTA |  |  | 1 |  |  |  | [14] |
| *SLC19A1* rs1051266, recessive model | 1 |  |  |  |  |  | [13] |
| *SLC19A1* rs2274808, minor allele |  |  | 1 |  |  |  | [12] |
| *SLC19A1* rs9977268, minor allele |  |  | 1 |  |  |  | [12] |
| *SLC19A1* rs11702425, minor allele |  |  | 1 |  |  |  | [12] |
| *SLC19A1* rs1556329, minor allele |  |  | 1 |  |  |  | [12] |
| *SLC19A1* rs2236475, minor allele |  |  | 1 |  |  |  | [12] |
| *SLC19A1* rs2236479, minor allele |  |  | 1 |  |  |  | [12] |
| *SLC19A1* rs7279445, minor allele |  |  | 1 |  |  |  | [12] |
| *SLC19A1* rs3753019, minor allele |  |  | 1 |  |  |  | [12] |
| *SLC19A1* rs10483080, minor allele |  |  | 1 |  |  |  | [12] |
| *SLC19A1* rs2838950, minor allele |  |  | 1 |  |  |  | [12] |
| *SLC19A1* rs2838951, minor allele |  |  | 1 |  |  |  | [12] |
| *SLC19A1* rs12483377, minor allele |  |  | 1 |  |  |  | [12] |
| *SLC19A1* rs7499, minor allele |  |  | 1 |  |  |  | [12] |
| *SLC19A1* rs17004785, minor allele |  |  | 1 |  |  |  | [12] |
| *SLC19A1* rs2838956, minor allele |  |  | 1 |  |  |  | [12] |
| *SLC19A1* rs3788205, minor allele |  |  | 1 |  |  |  | [12] |
| *SLC46A1* rs2239907, dominant model |  |  | 2 |  |  |  | [1] |
| *SLC46A1* rs2239907, recessive model |  |  | 1 |  |  |  | [13] |
| *FOLR2* rs514933, recessive model |  |  | 1 |  |  |  | [13] |
| *ADORA2A* rs5751876, dominant model |  |  | 2 |  |  |  | [1] |
| *ADORA2A* rs1041748, minor allele |  |  | 1 |  |  |  | [12] |
| *ADORA2A* rs5751846, minor allele |  |  | 1 |  |  |  | [12] |
| *ADORA2A* rs6004146, minor allele |  |  | 1 |  |  |  | [12] |
| *ADORA2A* rs5751862, minor allele |  |  | 1 |  |  |  | [12] |
| *ADORA2A* rs4822488, minor allele |  |  | 1 |  |  |  | [12] |
| *ADORA2A* rs3761422, minor allele |  |  | 1 |  |  |  | [12] |
| *ADORA2A* rs11704811, minor allele |  |  | 1 |  |  |  | [12] |
| *ADORA2A* rs2236624, minor allele |  |  | 1 |  |  |  | [12] |
| *THAP6* rs3853187, minor allele |  |  | 1 |  |  |  | [14] |
| *THAP6* rs1841934, minor allele |  |  | 1 |  |  |  | [14] |
| *THAP6* rs2126854, minor allele |  |  | 1 |  |  |  | [14] |
| *THAP6* rs9307834, minor allele |  |  | 1 |  |  |  | [14] |
| *THAP6* rs6535523, minor allele |  |  | 1 |  |  |  | [14] |
| *THAP6* rs12649508, minor allele |  |  | 1 |  |  |  | [14] |
| *BAT1* rs3132454, minor allele |  |  | 1 |  |  |  | [14] |
| *BAT1* rs9267464, minor allele |  |  | 1 |  |  |  | [14] |
| *BAT1* rs3093993, minor allele |  |  | 1 |  |  |  | [14] |
| *BAT1* rs3093992, minor allele |  |  | 1 |  |  |  | [14] |
| *BAT1* rs3093988, minor allele |  |  | 1 |  |  |  | [14] |
| *BAT1* rs2734574, minor allele |  |  | 1 |  |  |  | [14] |
| *BAT1* rs2259435, minor allele |  |  | 1 |  |  |  | [14] |
| *BAT1* rs3130055, minor allele |  |  | 1 |  |  |  | [14] |
| *BAT1* rs3219190, minor allele |  |  | 1 |  |  |  | [14] |
| *BAT1* rs2734583, minor allele |  |  | 1 |  |  |  | [14] |
| *BAT1* rs2239709, minor allele |  |  | 1 |  |  |  | [14] |
| *BAT1* rs2844509, minor allele |  |  | 1 |  |  |  | [14] |
| *BAT1* rs2239705, minor allele |  |  | 1 |  |  |  | [14] |
| *BAT1* rs2071591, minor allele |  |  | 1 |  |  |  | [14] |
| *BAT1* rs2523500, minor allele |  |  | 1 |  |  |  | [14] |
| *BAT1* rs6916921, minor allele |  |  | 1 |  |  |  | [14] |
| *BAT1* rs6929796, minor allele |  |  | 1 |  |  |  | [14] |
| *ZEB1* rs11008463, minor allele |  |  | 1 |  |  |  | [14] |
| *ZEB1* rs2839657, minor allele |  |  | 1 |  |  |  | [14] |
| *ZEB1* rs172683, minor allele |  |  | 1 |  |  |  | [14] |
| *MALAT1* rs600231, minor allele |  |  | 1 |  |  |  | [14] |
| *MALAT1* rs3200401, minor allele |  |  | 1 |  |  |  | [14] |
| *NFATC2IP* rs12931589, minor allele |  |  | 1 |  |  |  | [14] |
| *NFATC2IP* rs11150675, minor allele |  |  | 1 |  |  |  | [14] |
| *NFATC2IP* rs7192056, minor allele |  |  | 1 |  |  |  | [14] |
| Region *LMX1A, PBX1* |  |  |  | 0/1 |  |  | [15] |
| Region *CDH6* |  |  |  | 0/1 |  |  | [15] |
| Region *CFTR, CTTNBP2* |  |  |  | 0/1 |  |  | [15] |
| Region *CSMD1* |  |  |  | 0/1 |  |  | [15] |
| Region *SNX16* |  |  |  | 0/1 |  |  | [15] |
| Region *PVT1, ADCY8* |  |  |  | 0/1 |  |  | [15] |
| Region *ZMIZ1* |  |  |  | 0/1 |  |  | [15] |
| Region *ANGPTL5, KIAA1377* |  |  |  | 0/1 |  |  | [15] |
| Region *ANKS1B, ANO4, ARL1, SPIC* |  |  |  | 0/1 |  |  | [15] |
| Region *CMKLR1* |  |  |  | 0/1 |  |  | [15] |
| Region *GABRB3* |  |  |  | 0/1 |  |  | [15] |
| Region *TGIF1* |  |  |  | 0/1 |  |  | [15] |
| Region *CYTH4* |  |  |  | 0/1 |  |  | [15] |
| Region *CACNA1I* |  |  |  | 0/1 |  |  | [15] |
| **Abbreviations:** *ABCB1* to *ABCG2,* members of the adenosine triphosphate binding cassette (ABC) transporters; ACR70, American College of Rheumatology paediatric 70 response criterion; *ADCY8,* adenylate cyclase 8; *ADORA2A,* adenosine A2A receptor; ALT, alanine aminotransferase; *AMPD1,* adenosine monophosphate deaminase; ANA, antinuclear antibodies; *ANGPTL5,* angiopoietin-like 5; *ANKS1B,* ankyrin repeat and sterile alpha motif domain containing 1B; *ANO4,* anoctamin 4; *ARL1,* ADP-ribosylation factor-like 1; AST, aspartate aminotransferase; *ATIC,* 5-aminoimidazole-4-carboxamide ribonucleotide transformylase; *BAT1,* HLA-B associated transcript 1; *CACNA1I,* calcium-channel, voltage-dependent, T type, alpha 1I subunit; *CDH6,* cadherin 6; *CFTR,* cystic fibrosis transmembrane conductance regulator; CHAQ, childhood health assessment questionnaire; CHQ, child health questionnaire; *CMKLR1,* chemokine-like receptor 1; CRP, C-reactive protein; *CSMD1,* CUB and Sushi multiple domains 1; *CTTNPB2,* cortacting-binding protein 2; *CYTH4,* cytohesin 4; *DHFR,* dihydrofolate reductase; ESR, erythrocyte sedimentation rate; *FOLR,* folate receptor; *FPGS,* folylpolyglutamate synthetase; GA, global assessment; *GABRB3,* gamma-aminobutyric acid A receptor, beta 3; *GGH,* γ-glutamyl hydrolase; HLA, human leukocyte antigen; IFN, interferon; IL, interleukin; *ITPA,* inosine triphosphatase; JADAS, juvenile arthritis disease activity score; JIA, juvenile idiopathic arthritis; *LMX1A,* LIM homeobox transcription factor 1 alpha; *MALAT1,* metastasis associated lung adenocarcinoma transcript 1; MCP, metacarpophalangeal joint; MRP, myeloid-related protein; *MTHFD1,* methylenetetrahydrofolate dehydrogenase; *MTHFR,* methylenetetrahydrofolate reductase; *MTRR,* methionine synthase reductase; MTX, methotrexate; MTX-PG, methotrexate polyglutamate; *NFATC2IP,* nuclear factor of activated T-cells, cytoplasmic, calcineurin-dependent 2 interacting protein; NSAIDs, non-steroidal anti-inflammatory drugs; *PBX1,* pre-B-cell leukemia homeobox 1; PGA, physician’s global assessment; PhS, physical component summary score; PIP, proximal interphalangeal joint; PsS, psychosocial component summary score; *PVT1,* Pvt1 oncogene; *SHMT1,* serine hydroxymethyltransferase; *SLC,* solute carrier; SNP, single nucleotide polymorphism; *SNX16,* sorting nexin 16; *SPIC,* Spi-C transcription factor; *TGIF1,* transforming growth factor beta-induced factor homeobox 1; *THAP6,* THAP domain containing 6; TNF, tumor necrosis factor; *TYMS,* thymidylate synthase; VAS, visual analog score; *ZEB1,* zinc finger-enhancer protein 1, *ZMIZ1,* zinc-finger, MIZ-type containing 1.  **^a^** Values are the number of studies which found that particular result for the respective predictors; **^b^** The first number in this column refers to the number of studies which performed a multivariate analysis disconfirming the univariate finding (studies in which the multivariate analysis confirmed the univariate finding, are shown in the columns labelled -- and +, respectively). The second number in this column refers to the number of studies which did not perform a multivariate analysis; **^c^** In studies that evaluated onset categories oligoarticular, polyarticular and systemic, results have been duplicated for oligoarthritis persistent and extended, and polyarthritis RF positive and negative respectively; **^d^** SNPs were analyzed in different ways, denoted in the table as follows: minor allele: allelic comparison. Odds ratios shown in the source study are allelic odds ratios, indicating the increase in odds per minor allele. Dominant model: homozygous variant and heterozygous subjects vs. wild type. Recessive model: homozygous variant vs. heterozygous and wild type. Haplotype: recessive model: subjects homozygous for the shown haplotype versus all other subjects.  **Symbols used:**  -- lower chance of favourable outcome in multivariate analysis (p ≤ 0.05)  < lower chance of favourable outcome in univariate analysis (p ≤ 0.05)  NS: not significant (p > 0.05)  S: significant in univariate analysis. Direction of the effect not shown, or different SNPs in the same region with opposed effects (p ≤ 0.05)  > higher chance of favourable outcome in univariate analysis (p ≤ 0.05)  + higher chance of favourable outcome in multivariate analysis (p ≤ 0.05) | | | | | | | |

Reference List

1. Bulatovic M, Heijstek MW, Van Dijkhuizen EHP, Wulffraat NM, Pluijm SM, de Jonge R: **Prediction of clinical non-response to methotrexate treatment in juvenile idiopathic arthritis.** *Ann Rheum Dis* 2012, **71:**1484-1489.

2. Moncrieffe H, Ursu S, Holzinger D, Patrick F, Kassoumeri L, Wade A, Roth J, Wedderburn LR: **A subgroup of juvenile idiopathic arthritis patients who respond well to methotrexate are identified by the serum biomarker MRP8/14 protein.** *Rheumatology (Oxford)* 2013, **52:**1467-1476.

3. Tukova J, Chladek J, Hroch M, Nemcova D, Hoza J, Dolezalova P: **677TT genotype is associated with elevated risk of methotrexate (MTX) toxicity in juvenile idiopathic arthritis: treatment outcome, erythrocyte concentrations of MTX and folates, and MTHFR polymorphisms.** *J Rheumatol* 2010, **37:**2180-2186.

4. Ravelli A, Viola S, Migliavacca D, Ruperto N, Pistorio A, Martini A: **The extended oligoarticular subtype is the best predictor of methotrexate efficacy in juvenile idiopathic arthritis.** *J Pediatr* 1999, **135:**316-320.

5. Bartoli M, Taro M, Magni-Manzoni S, Pistorio A, Traverso F, Viola S, Magnani A, Gasparini C, Martini A, Ravelli A: **The magnitude of early response to methotrexate therapy predicts long-term outcome of patients with juvenile idiopathic arthritis.** *Ann Rheum Dis* 2008, **67:**370-374.

6. Cespedes-Cruz A, Gutierrez-Suarez R, Pistorio A, Ravelli A, Loy A, Murray KJ, Gerloni V, Wulffraat N, Oliveira S, Walsh J et al.: **Methotrexate improves the health-related quality of life of children with juvenile idiopathic arthritis.** *Ann Rheum Dis* 2008, **67:**309-314.

7. Vilca I, Munitis PG, Pistorio A, Ravelli A, Buoncompagni A, Bica B, Campos L, Hafner R, Hofer M, Ozen S et al.: **Predictors of poor response to methotrexate in polyarticular-course juvenile idiopathic arthritis: analysis of the PRINTO methotrexate trial.** *Ann Rheum Dis* 2010, **69:**1479-1483.

8. Wallace CA, Sherry DD, Mellins ED, Aiken RP: **Predicting remission in juvenile rheumatoid arthritis with methotrexate treatment.** *J Rheumatol* 1993, **20:**118-122.

9. Masi L, Ricci L, Zulian F, Del Monte F, Simonini G, Capannini S, De Martino M, Brandi ML, Falcini F: **Serum osteopontin as a predictive marker of responsiveness to methotrexate in juvenile idiopathic arthritis.** *J Rheumatol* 2009, **36:**2308-2313.

10. Klein A, Kaul I, Foeldvari I, Ganser G, Urban A, Horneff G: **Efficacy and safety of oral and parenteral methotrexate therapy in children with juvenile idiopathic arthritis: an observational study with patients from the German Methotrexate Registry.** *Arthritis Care Res (Hoboken )* 2012, **64:**1349-1356.

11. Bulatovic Calasan M, den Boer E, de Rotte MC, Vastert SJ, Kamphuis S, de Jonge R, Wulffraat NM: **Methotrexate polyglutamates in erythrocytes are associated with lower disease activity in juvenile idiopathic arthritis patients.** *Ann Rheum Dis* 2013.

12. Hinks A, Moncrieffe H, Martin P, Ursu S, Lal S, Kassoumeri L, Weiler T, Glass DN, Thompson SD, Wedderburn LR et al.: **Association of the 5-aminoimidazole-4-carboxamide ribonucleotide transformylase gene with response to methotrexate in juvenile idiopathic arthritis.** *Ann Rheum Dis* 2011, **70:**1395-1400.

13. de Rotte MC, Bulatovic M, Heijstek MW, Jansen G, Heil SG, van Schaik RH, Wulffraat NM, de Jonge R: **ABCB1 and ABCC3 gene polymorphisms are associated with first-year response to methotrexate in juvenile idiopathic arthritis.** *J Rheumatol* 2012, **39:**2032-2040.

14. Moncrieffe H, Hinks A, Ursu S, Kassoumeri L, Etheridge A, Hubank M, Martin P, Weiler T, Glass DN, Thompson SD et al.: **Generation of novel pharmacogenomic candidates in response to methotrexate in juvenile idiopathic arthritis: correlation between gene expression and genotype.** *Pharmacogenet Genomics* 2010, **20:**665-676.

15. Cobb J, Cule E, Moncrieffe H, Hinks A, Ursu S, Patrick F, Kassoumeri L, Flynn E, Bulatovic M, Wulffraat N et al.: **Genome-wide data reveal novel genes for methotrexate response in a large cohort of juvenile idiopathic arthritis cases.** *Pharmacogenomics J* 2014.
