# Supplementary material for: Prediction of methotrexate efficacy and adverse events in patients with juvenile idiopathic arthritis: a systematic literature review
Source: Pediatr Rheumatol Online J. 2014 Dec 11;12:51. doi: 10.1186/1546-0096-12-51 (PMC4269851; doi:10.1186/1546-0096-12-51)
Supplement: Supplementary file 2 — Additional file 2: Table S2: Results for outcome absence of MTX adverse eventsa. (DOCX 116 KB) [file 12969_2014_2173_MOESM2_ESM.docx]

| **Additional file 2: Table S2.** Results for outcome absence of MTX adverse events**^a^** | | | | | | |
| --- | --- | --- | --- | --- | --- | --- |
| Baseline predictors (within 6 months after MTX start) | Number of studies | | | | | References |
|  | -- | <**^b^** | NS | >**^b^** | + |  |
| *Demographics* |  |  |  |  |  |  |
| Gender: female |  |  | 3 |  |  | [1-3] |
| Higher age at onset |  |  | 3 |  |  | [1-3] |
| Higher age at MTX start |  |  | 2 |  |  | [1,2] |
| Longer disease duration before MTX start |  |  | 3 |  |  | [1-3] |
|  |  |  |  |  |  |  |
| *JIA category****^c^*** |  |  |  |  |  |  |
| Oligoarticular persistent |  |  |  |  |  |  |
| Oligoarticular extended |  |  | 1 |  |  | [1] |
| Systemic |  |  | 2 |  |  | [1,2] |
| Polyarticular, rheumatoid factor negative | 1 |  | 1 |  |  | [1,2] |
| Polyarticular, rheumatoid factor positive | 1 |  | 1 |  |  | [1,2] |
| Psoriatic |  |  | 1 |  |  | [2] |
| Enthesitis-related arthritis |  |  | 1 |  |  | [2] |
| Undifferentiated |  |  | 1 |  |  | [2] |
|  |  |  |  |  |  |  |
| *Disease activity* |  |  |  |  |  |  |
| Higher active joint count |  |  | 2 |  |  | [1,2] |
| Higher limited joint count |  |  | 1 |  |  | [2] |
| Higher PGA |  |  | 1 |  |  | [2] |
| Higher parent/patient GA |  |  | 1 |  |  | [2] |
| Higher pain VAS |  |  | 1 |  |  | [2] |
| Higher CHAQ score |  |  | 2 |  |  | [1,2] |
| Higher JADAS-27 |  |  |  | 1/0 |  | [2] |
| Hand or wrist involvement |  |  | 1 |  |  | [1] |
| Uveitis present |  |  | 1 |  |  | [2] |
|  |  |  |  |  |  |  |
| *Laboratory* |  |  |  |  |  |  |
| ANA positive |  |  | 1 |  |  | [2] |
| Rheumatoid factor positive |  |  | 1 |  |  | [2] |
| Higher ESR |  |  | 2 |  |  | [1,2] |
| Higher CRP |  |  | 1 |  |  | [2] |
| Higher hemoglobin |  |  | 1 |  |  | [2] |
| Higher leukocyte count |  |  | 1 |  |  | [2] |
| Higher thrombocyte count |  |  | 1 |  | 1 | [1,2] |
| Higher ALT level |  |  |  |  | 1 | [2] |
| Higher AST level |  |  | 1 |  |  | [2] |
| Higher creatinine level |  |  | 1 |  |  | [2] |
|  |  |  |  |  |  |  |
| *Medication* |  |  |  |  |  |  |
| Subcutaneous route of administration |  |  | 1 |  |  | [4] |
| Restart of MTX |  |  | 1 |  |  | [2] |
| Start with folic acid |  |  | 1 |  |  | [3] |
| Taking corticosteroids |  |  | 2 |  |  | [1,3] |
| Taking NSAIDs |  |  | 1 |  |  | [2] |
|  |  |  |  |  |  |  |
| *MTX-polyglutamates* |  |  |  |  |  |  |
| Higher MTX-PG1 |  |  | 1 |  |  | [5] |
| Higher MTX-PG2 |  |  | 1 |  |  | [5] |
| Higher MTX-PG3 |  |  | 1 |  |  | [5] |
| Higher MTX-PG4 |  |  | 1 |  |  | [5] |
| Higher MTX-PG5 |  |  | 1 |  |  | [5] |
| Higher MTX-PG3-5 |  |  | 1 |  |  | [5] |
| Higher total MTX-PG |  |  | 1 |  |  | [5] |
|  |  |  |  |  |  |  |
| *Imaging* |  |  |  |  |  |  |
| Presence of radiologic lesions |  |  | 1 |  |  | [1] |
|  |  |  |  |  |  |  |
| *Genetics****^d^*** |  |  |  |  |  |  |
| HLA-B27 positive |  |  | 1 |  |  | [2] |
| *MTHFR* rs1801133, dominant model |  | 0/1 | 1 |  |  | [3,6] |
| *MTHFR* rs1801133, recessive model |  | 1/0 | 2 |  |  | [2,3,7] |
| *MTHFR* rs1801133, homozygous variant vs. wild type |  | 1/0 |  |  |  | [7] |
| *MTHFR* rs1801133, heterozygous vs. wild type |  | 0/1 | 1 |  |  | [6,7] |
| *MTHFR* rs1801133, minor allele | 1 |  | 1 |  |  | [3,7] |
| *MTHFR* rs1801131, dominant model |  |  | 4 |  |  | [2,3,6,7] |
| *MTHFR* rs1801131, recessive model |  |  | 1 |  |  | [3] |
| *MTHFR* rs1801131, homozygous variant vs. wild type |  |  | 1 |  |  | [7] |
| *MTHFR* rs1801131, heterozygous vs. wild type |  |  | 2 |  |  | [6,7] |
| *MTHFR* rs1801131, minor allele |  |  | 2 |  |  | [3,7] |
| *MTRR* rs1801394, dominant model |  |  | 1 |  |  | [2] |
| *AMPD1* rs17602729, dominant model |  |  | 1 |  |  | [2] |
| *ATIC* rs2372536, dominant model |  |  | 2 |  |  | [2,3] |
| *ATIC* rs2372536, recessive model |  |  | 1 |  |  | [3] |
| *ATIC* rs2372536, minor allele |  |  | 1 |  |  | [3] |
| *ABCB1* rs1128503, recessive model |  |  | 1 |  |  | [2] |
| *ABCB1* rs1045642, recessive model |  |  | 1 |  |  | [2] |
| *ABCB1* rs2032582, dominant model |  |  | 1 |  |  | [2] |
| *ABCC1* rs35592, dominant model |  |  | 1 |  |  | [2] |
| *ABCC1* rs3784862, dominant model |  |  | 1 |  |  | [2] |
| *ABCC2* rs4148396, recessive model |  |  | 1 |  |  | [2] |
| *ABCC2* rs717620, dominant model |  |  | 1 |  |  | [2] |
| *ABCC3* rs4793665, dominant model |  |  | 1 |  |  | [2] |
| *ABCC3* rs3785911, dominant model |  |  | 1 |  |  | [2] |
| *ABCC4* rs868853, dominant model |  |  | 1 |  |  | [2] |
| *ABCC4* rs2274407, dominant model |  |  | 1 |  |  | [2] |
| *ABCC5* rs2139560, dominant model |  |  | 1 |  |  | [2] |
| *ABCG2* rs13120400, dominant model |  |  | 1 |  |  | [2] |
| *ABCG2* rs2231142, dominant model |  |  | 2 |  |  | [2,3] |
| *ABCG2* rs2231142, recessive model |  |  | 1 |  |  | [3] |
| *ABCG2* rs2231142, minor allele |  |  | 1 |  |  | [3] |
| *GGH* rs1800909, dominant model |  |  | 1 |  |  | [3] |
| *GGH* rs1800909, recessive model |  |  |  |  | 1 | [3] |
| *GGH* rs1800909, minor allele |  |  | 1 |  |  | [3] |
| *GGH* rs11545078, recessive model |  |  | 1 |  |  | [3] |
| *GGH* rs11545078, minor allele |  |  | 1 |  |  | [3] |
| *GGH* rs10106587, dominant model |  |  | 1 |  |  | [2] |
| *GGH* rs3758149, dominant model |  |  | 1 |  |  | [2] |
| *FPGS* rs10106, dominant model |  |  | 1 |  |  | [3] |
| *FPGS* rs10106, recessive model |  |  | 1 |  |  | [3] |
| *FPGS* rs10106, minor allele |  |  | 1 |  |  | [3] |
| *FPGS* rs4451422, dominant model |  |  | 1 |  |  | [2] |
| *ITPA* rs1127354, recessive model |  |  | 1 |  |  | [2] |
| *SLC19A1* rs1051266, dominant model |  |  | 1 |  |  | [3] |
| *SLC19A1* rs1051266, recessive model |  |  | 2 |  |  | [2,3] |
| *SLC19A1* rs1051266, minor allele |  |  | 1 |  |  | [3] |
| *SLC46A1* rs2239907, dominant model |  |  | 1 |  |  | [2] |
| *ADORA2A* rs5751876, recessive model |  |  | 1 |  |  | [2] |
| For declaration of symbols, footnotes and abbreviations, see table 4. | | | | | | |

Reference List

1. Ravelli A, Viola S, Migliavacca D, Ruperto N, Pistorio A, Martini A: **The extended oligoarticular subtype is the best predictor of methotrexate efficacy in juvenile idiopathic arthritis.** *J Pediatr* 1999, **135:**316-320.

2. Van Dijkhuizen EHP, Bulatovic Calasan M, Pluijm SMF, De Rotte MCFJ, Vastert SJ, Kamphuis SMM, de Jonge R, Wulffraat NM. **Prediction of Methotrexate Intolerance in Juvenile Idiopathic Arthritis: a prospective, observational cohort study.** Pediatr Rheumatol Online J submitted. 2014.

3. Yanagimachi M, Naruto T, Hara T, Kikuchi M, Hara R, Miyamae T, Imagawa T, Mori M, Kaneko T, Morita S et al.: **Influence of polymorphisms within the methotrexate pathway genes on the toxicity and efficacy of methotrexate in patients with juvenile idiopathic arthritis.** *Br J Clin Pharmacol* 2011, **71:**237-243.

4. Klein A, Kaul I, Foeldvari I, Ganser G, Urban A, Horneff G: **Efficacy and safety of oral and parenteral methotrexate therapy in children with juvenile idiopathic arthritis: an observational study with patients from the German Methotrexate Registry.** *Arthritis Care Res (Hoboken )* 2012, **64:**1349-1356.

5. Bulatovic Calasan M, den Boer E, de Rotte MC, Vastert SJ, Kamphuis S, de Jonge R, Wulffraat NM: **Methotrexate polyglutamates in erythrocytes are associated with lower disease activity in juvenile idiopathic arthritis patients.** *Ann Rheum Dis* 2013.

6. Schmeling H, Biber D, Heins S, Horneff G: **Influence of methylenetetrahydrofolate reductase polymorphisms on efficacy and toxicity of methotrexate in patients with juvenile idiopathic arthritis.** *J Rheumatol* 2005, **32:**1832-1836.

7. Tukova J, Chladek J, Hroch M, Nemcova D, Hoza J, Dolezalova P: **677TT genotype is associated with elevated risk of methotrexate (MTX) toxicity in juvenile idiopathic arthritis: treatment outcome, erythrocyte concentrations of MTX and folates, and MTHFR polymorphisms.** *J Rheumatol* 2010, **37:**2180-2186.
